# A ‐1573T>C SNP within the human TRAIL promoter determines TRAIL expression and HCC tumor progression

**DOI:** 10.1002/cam4.854

**Published:** 2016-08-31

**Authors:** Katja Piras‐Straub, Khaleda Khairzada, Peri Kocabayoglu, Andreas Paul, Guido Gerken, Kerstin Herzer

**Affiliations:** ^1^ Department of General, Visceral, and Transplantation SurgeryUniversity Hospital GermanyEssenGermany; ^2^ Department of Gastroenterology and HepatologyUniversity Hospital EssenEssenGermany

**Keywords:** Apoptosis, hepatocellular carcinoma, liver resection, liver transplantation, TRAIL

## Abstract

The cytokine tumor necrosis factor (TNF)‐related apoptosis‐inducing ligand (TRAIL) induces apoptosis in liver cancer cells but not in normal liver cells. Therefore, TRAIL got credited to play a role in hepatocellular carcinoma (HCC) development and progression. Impaired expression of TRAIL in HCC cells and sequence variations in the TRAIL promoter may facilitate development, growth, and spread . The TRAIL promoter was sequenced from liver tissue of 93 patients undergoing partial liver resection (PRT) or liver transplantation (LT) for HCC. TRAIL mRNA expression was investigated by quantitative real‐time PCR. A variant ‐1573T>C (single‐nucleotide polymorphism; C, cytosine) SNP was characterized by electron mobility shift assay and supershift assays. Functionality of the ‐1573T>C SNP was analyzed in reporter gene assays and cell migration assays. In approximately 30% of HCC samples, a loss‐of‐function shift of the binding pattern due to a ‐1573T>C SNP was found within the human TRAIL promoter. Correlation analysis revealed significantly lower TRAIL expression in HCC samples with the ‐1573C sequence (*P *≤* *0.05). Reporter gene assays revealed significantly reduced inducibility of the TRAIL promoter due to the ‐1573C sequence. The variant ‐1573C sequence impaired not only binding of transcription factors but also expression of TRAIL. Interestingly, this impairment resulted in enhanced migration activity and colony formation of the liver tumor cells. Our findings suggest that loss of function of the human TRAIL promoter due to the ‐1573T>C SNP leads to reduced expression and impaired inducibility of TRAIL, with the consequence of enhanced growth and migration of tumor cells, ultimately resulting in the progression of the HCC.

## Introduction

Hepatocellular carcinoma (HCC) is a severe health burden, as it is one of the most common cancers worldwide and one of the first three causes of cancer‐related mortality 1, 2. Although the incidence of HCC caused by hepatitis C (HCV)‐related liver cirrhosis is expected to decrease because of more efficient therapeutic options 3, 4, this decrease will be balanced by a cumulative burden of alcoholic steatohepatitis (ASH) or nonalcoholic steatohepatitis (NASH) as precancerous states 5. Curative treatment options are limited to liver transplantation or liver resection, whereas local ablative therapies are successful for only a limited number of patients and systemic chemotherapy is not a viable option, primarily because of its molecular heterogeneity 6, 7, 8. Although liver transplantation for HCC is characterized by a good long‐term outcome, the tumor recurs in a certain percentage of patients 9. Therefore, there is an obvious need for a deeper understanding of the molecular mechanisms of development and growth, with the aim of defining novel therapeutic agents and strategies, as well as predictors of outcome. The molecular process of HCC development implies the activation of oncogenes and the repression of apoptosis; but, the stepwise process of genetic alteration is also observable 10, 11, 12, 13.

Because of experimental evidence that the tumor necrosis factor‐related apoptosis‐inducing ligand (TRAIL) induces apoptosis in liver cancer cells but not in healthy hepatocytes, this molecule was suggested to have potential as a promising alternative or additive therapeutic tool for HCC 14. The correlation between TRAIL receptor expression, poor prognosis, and tumor recurrence has previously been shown 15, 16. The influence of TRAIL on the aggressiveness of tumor growth has been indicated by in vivo analyses demonstrating that TRAIL knockout mice exhibit enhanced formation of metastases 17. Although the influences of the TRAIL system on the pathogenesis of HCC are not completely understood, recent studies have demonstrated that TRAIL expression levels predict the growth and recurrence of HCC and that TRAIL expression is impaired in human HCCs 18. This evidence suggests that the regulation of TRAIL expression plays a role in the development and growth of HCC. Previous studies have shown the importance of SNPs (single‐nucleotide polymorphism) present along the TRAIL gene for carcinogenesis and fatty liver diseases 19, 20, but until now no investigations concerning HCC were performed.

To further elucidate the molecular mechanism of the impaired TRAIL regulation, we analyzed a large number of HCC samples and surrounding noncancerous liver tissues from patients undergoing liver resection or liver transplantation. We found that the loss of TRAIL is not only a common feature of advanced HCC but is caused by the ‐1573T>C SNP within an in silico GATA‐1binding site in the human TRAIL promoter.

## Material and Methods

### Tissues

The liver tissues from 93 HCC patients were included in this analysis. The tumor tissue as well as the surrounding noncancerous liver tissue were collected after partial hepatectomy or liver transplantation. All human tissue samples were collected in accordance with the Declaration of Helsinki and patients provided informed consent for the scientific use of liver tissue.

### Assessment of tumors and surrounding noncancerous liver tissue samples

Tumors were graded according to the World Health Organization (WHO) criteria. For this study, identification of the surrounding noncancerous liver tissues as well as the grading of the tumor was assessed by a pathologist. The tumor grading was based on the tissue area with the highest grade.

### mRNA isolation and quantitative real‐time PCR

For TRAIL mRNA analysis, 30 mg of HCC tissue (TT) as well as 30 mg surrounding noncancerous liver tissue (TST) were macrodissected and lysed in QIAzol Lysis Reagent (Qiagen, Venlo, Netherlands). For RNA isolation, the RNeasy Mini Kit (Qiagen) was used according the manufacturer's instructions. The QuantiFast SYBR Green RT‐PCR Kit (Qiagen) with a combined protocol for cDNA synthesis and subsequent RT‐PCR were used with the following cycling conditions: reverse transcription at 50°C for 10 min, initial PCR activation step at 95°C for 5 min, followed by 40 cycles including a 10‐sec denaturation at 95°C and a combined annealing and extension step for 30 sec at 60°C. TRAIL was detected by the Hs_TNFSF10_1_SG QuantiTect Primer Assay (QT00079212; Qiagen) and normalized to the expression of actin as internal control (*β‐actin* gene [ACTB]; Eurofins MWG Operon: forward primer, 5′‐tccctggagaagagctacga‐3′; reverse primer, 5′‐agcactgtgttggcgtacag‐3′).

### Sequencing of the human TRAIL promoter region

For genomic DNA isolation, 25 mg of TT and surrounding TST were macrodissected and lysed in ALT buffer (Qiagen). After digestion with proteinase K and RNAse A, genomic DNA was isolated with the QIAamp DNA Mini Kit (Qiagen) according to the manufacturer's instructions. The TRAIL promoter region was amplified with the HotStarTaq Plus Master Mix Kit (Qiagen) and two primers: TRAIL fwd, cttgacctgaccccgagata, and TRAIL rev, cccacagagaaaggaagcag, according to the manufacturer's instructions. The complete sequence was analyzed by SimpleSeq. (Eurofins MWG Operon) with the following sequencing primers: fwd 1, tccactgccagaaactctga; fwd 2, ggcatgttgtcttggtaggg; fwd 3, cttgacctgaccccgagata; rev 1, gctactgtgagggtgggaag.

### Cloning of luciferase reporter gene constructs of the human TRAIL promoter

Firefly luciferase reporter gene constructs with the repetitive sequence area around the GATA‐1‐binding site (pGL3 basic ‐1573T, intact GATA‐1‐binding site; pGL3 basic ‐1573C, no GATA‐1‐binding site) were constructed by cloning synthetic sequences (‐1573T, ctttacagatagataagacacgacttattttacagatagataagacacgacttattttacagatagataagacacgacttattttacaga tagataagacacgacttattttacagatagataagacacgacttattttacagatagataagacacgacttattttacagatagataagacacgacttattttacagatagataagacacgacttata; ‐1573C, ctttacagacagataagacacgacttattttacagacagata agacacgacttattttacagacagataagacacgacttattttacagacagataagacacgacttattttacagacagataagacacgacttattttacagacagataagacacgacttattttacagacagataagacacgacttattttacagacagataagacacgacttata) between the KpnI and HindIII restriction sites of the pGL3 basic vector (Promega, Madison, WI).

The firefly luciferase reporter gene construct containing the complete TRAIL promoter region, including the intact GATA‐1‐binding site (pGL3 basic TRAIL promoter [TP] complete ‐1573T), was constructed by amplifying the TRAIL promoter sequence of the hepatoma cell line, Huh7 (primer fwd, gcgcggtacccggttatcctactcatctttc; primer rev, gcgcaagctttcctgtcagagtctgactgc; Eurofins MWG Operon) and inserting the sequence between the KpnI and HindIII restriction sites of the pGL3 basic vector (Promega). The firefly luciferase reporter gene construct with the complete TRAIL promoter region lacking the GATA‐1‐binding site (pGL3 basic TP complete ‐1573C) was constructed by site‐directed mutagenesis of the pGL3 basic TP complete ‐1573T construct with two primers: fwd, 5′‐ phosphorylated [PHO]‐aggaagtgatggtgaccagc; rev, 5′‐PHO‐gcccttgccttctgtctgtaaatgag, and the Phusion Site‐Directed Mutagenesis Kit (Thermo Scientific, Waltham, Massachusetts, USA).

### Cell culture

Huh7 and HepG2 cells were obtained from the American Type Culture Collection (Rockville, Maryland, USA). Both cell lines were cultured in Dulbecco's modified essential medium (Life Technologies, Carlsbad, California, USA) supplemented with 10% heat‐inactivated fetal calf serum (Life Technologies) and 100 U/mL penicillin and 100 *μ*g/mL streptomycin sulfate (Life Technologies) in a humidified incubator with 5 % CO_2_ at 37°C.

For experimental conditions, Interferon (IFN)‐alpha (IFN‐*α*), IFN‐gamma (IFN‐*γ*), N'‐hydroxy‐N‐phenyloctanediamide (SAHA) as well as ionomycin and phorbol 12‐myristate 13‐acetate (PMA) were obtained from Sigma‐Aldrich (St. Louis, Missouri, USA) and dissolved as described by manufacturer's instructions.

### Transient transfection and luciferase assay

The transient transfections of Huh7 cells were performed with Fugene HD Transfection Reagent (Promega). For luciferase assays, 1 × 10^6^ Huh7 cells were seeded on 6‐well plates 1 day before transfection. Transfection efficiency was determined by cotransfection of 2 *μ*g of the reporter gene constructs with 100 ng of the pRL‐SV40 renilla luciferase expression vector (Promega). Cells were harvested 24 h after transfection, seeded on 96‐well plates, and stimulated as indicated in Figure 3. TRAIL‐inducing agents with known TRAIL‐inducing potential were used as indicated in Figure 3 21, 22, 23, 24. Cells were harvested and assayed with the Dual‐Glo Luciferase Assay System (Promega) according to the manufacturer's instructions, with a FLUOstar Omega Microplate Reader (BMG Labtech, Ortenberg, Germany).

### Extraction of nuclear protein extracts

Huh7 cells were cultured in 10 cm^2^ until 90% confluency was reached; they were then stimulated with 500 IU/mL IFN‐*α* (Sigma) or 1000 U/mL IFN‐*γ* (Sigma). After treatment for 6 h, cells were harvested with Trypsin‐EDTA (Life technologies), and the nuclear protein extracts were isolated with the NE‐PER Nuclear and Cytoplasmic Extraction Kit (Life technologies) according to the manufacturer's instructions.

### Electrophoretic mobility shift assay

Complementary single‐stranded oligonucleotides were synthesized (Eurofins MWG Operon) to span 24 bp on either site of the variant nucleotide (T/C) at position ‐1573 upstream of the transcription start site of the human TRAIL promoter. The 5′‐end of the sense oligonucleotide (‐1573T forward, 5′ biotinylated (BIO)‐cttaaatgtagactcatttacagaTagaaggcaagggcaggaagtgatg‐3′; reverse, 5′‐catcacttcctgcccttgccttctAtc tgtaaatgagtctacatttaag‐3′; ‐1573C forward, 5′BIO‐cttaaatgtagactcatttacagaCagaaggcaaggg caggaagtgatg‐3′; reverse, 5′‐catcacttcctgcccttgccttctGtctgtaaatgagtctacatttaag‐3′). The double‐stranded probes were annealed by heating the complementary oligonucleotides at 100°C for 10 min and then slowly cooling them to 4°C. An electron mobility shift assay (EMSA) was performed with 100 fmol of both ‐1573T/C sequence variants with or without the appropriate unlabeled competitor oligonucleotide with an excess of 200‐fold. The binding assay was performed with the LightShift Chemiluminescent EMSA Kit (Thermo Scientific) according to the manufacturer's instructions, with 3 *μ*L of nuclear protein extracts. For supershift assay, 4 *μ*g of GATA‐1 antibody (H‐200, Santa Cruz Biotechnology) was added to the EMSA reactions with the ‐1573T probe and nuclear extracts of Huh7 cells.

### siRNA transfection, migration assay, and colony‐formation assay

The RNA interference analysis was performed by transfecting Huh7 cells with small interfering RNA (siRNA) (FlexiTube siRNA Premix, Qiagen). One day before transfection, 1 × 10^6^ Huh7 cells were seeded on 6‐well plates. Huh7 cells were transfected with 25 nmol/L TRAIL‐specific siRNA (SI00056154) or control siRNA (SI03650318).

For the migration assay, 48 h after transfection, cells were harvested and 1 × 10^4^ cells were seeded in transwell inserts with a pore size of 8 *μ*m in serum‐free media with normal growth media in the outer chamber. After 24 h, the cells were fixed in 4% Roti^®^‐Histofix (Carl Roth, Karlsruhe, Germany) and stained with crystal violet. The cells were counted with a light microscope (Advanced Microscopy Group, Bothell, Washington, USA).

For the colony‐formation assay performed 48 h after transfection, cells were harvested and 1 × 10^4^ cells were seeded in a growth medium containing 0.35% agarose in 12‐well plates. During the 21‐day cultivation period, cell culture medium was changed twice per week. Staining was performed with 0.005% crystal violet (Sigma) for 2 h before the colonies were counted with a light microscope (Advanced Microscopy Group).

### Statistical analyses

Gene expression data for the HCC samples are expressed as mean plus or minus standard error of the mean (SEM) or as median and range. Promoter activities are expressed as relative light units (RLU): either as the ratio of firefly luciferase activity to renilla luciferase activity or as the ratio of stimulated cells to nonstimulated control cells. The influence of the ‐1573T>C SNP on the inducibility of the human TRAIL promoter was determined with a two‐tailed Student's *t*‐test. The binding pattern of the TRAIL promoter sequence is expressed as the relative light intensity (RLI) of the shift: specifically, the densitometric intensity ratio of the band shift to the densitometric intensity of the nonshifted probe for each condition. The fold change was calculated by normalizing the intensity ratio of the EMSA reactions after IFN‐*α* or IFN‐*γ* treatment with the ‐1573T or ‐1573C probe toward the intensity ratio of the EMSA reaction of untreated Huh7 nuclear extracts with the ‐1573T probe. Statistical analysis was performed with the two‐tailed Student's *t*‐test. The differences in TRAIL mRNA expression by human liver cells depend on nucleotide sequences within the GATA‐1‐binding site of the human TRAIL promoter; these differences were analyzed with the Mann–Whitney *U* Test. The correlation between ‐1573T>C SNP distribution and tumor grade is displayed as the percentage of tissue samples with the ‐1573C genotype. These differences were analyzed with the chi‐squared test with Pearson approximation. For all tests, statistical significance was set at the level of *P *≤* *0.05.

## Results

### The human TRAIL promoter sequence

**Figure 1:**
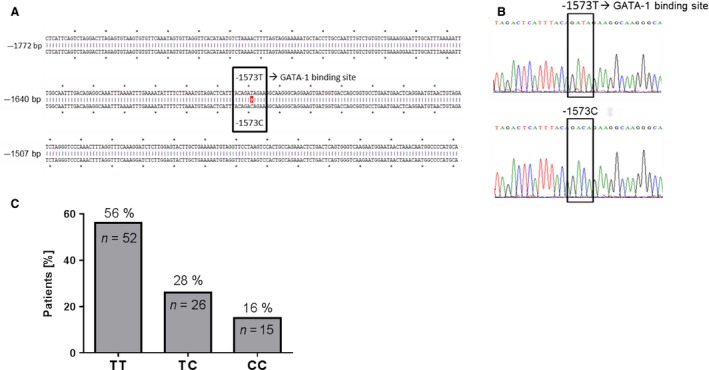
(A) Alignment of the TRAIL promoter sequence area (‐1772 bp to ‐1372 bp upstream of the transcription start site) spanning the in silico GATA‐1‐binding site (‐1573 bp upstream of the transcription start site). The sequence origins of tumor tissue containing a functional GATA‐1‐binding site (top) and tumor tissue containing an impaired GATA‐1‐binding site (bottom) in the TRAIL promoter sequence are also shown. (B) Chromatogram of sections around the GATA‐1‐binding site of the TRAIL promoter with a functional GATA‐1‐binding site (top) and with the ‐1573C variation (bottom) within the human TRAIL promoter. (C) Number of patients with analyzed TRAIL promoter sequence and the ratio of patients with a homozygous‐intact GATA‐1‐binding site (TT), a heterozygous‐impaired GATA‐1‐binding site (TC), or a homozygous‐impaired GATA‐1‐binding site (CC). T, thymine; C, cytosine; TRAIL, tumor necrosis factor‐related apoptosis‐inducing ligand.

The sequence of the TRAIL promoter was characterized in a large series of liver tissues from 93 HCC patients and 43 corresponding noncancerous liver tissues. The DNA sequences (corresponding to ‐1700 to ‐1 of the transcription start site) of the human TRAIL promoter were analyzed in silico for sequence variations, particularly in functional transcription factor binding sites. The nucleotide alignments demonstrate the incidence of a sequence variation at position ‐1573 upstream of the transcription start site of the human TRAIL promoter (Fig. 1A), identified below as the ‐1573T>C SNP which is located within an in silico identified GATA‐1 transcription factor binding site of the human TRAIL promoter. The nucleotide exchange from T to C at this position results in the loss of this GATA‐1‐binding site, referred to below as the ‐1573C SNP (Fig. 1B). Multiple alignments and more detailed analyses of all sequences demonstrated that nearly half of the analyzed HCC patients harbored at least the heterozygous ‐1573C genotype in the TRAIL promoter and, thus, lacked the functional in silico GATA‐1‐binding site (Fig. 1C). Most (56%) patients showed the homozygous ‐1573TT genotype at position ‐1573 of the TRAIL promoter in the HCC tissue with a functional in silico GATA‐1‐binding site, whereas 28% of the patients exhibited a heterozygous genotype (‐1573 TC) at this position and only 16% of the patients showed the homozygous ‐1573 CC genotype with the complete loss‐of‐function mutation.

### ‐1573T>C genotype in HCC tissues

**Figure 2:**
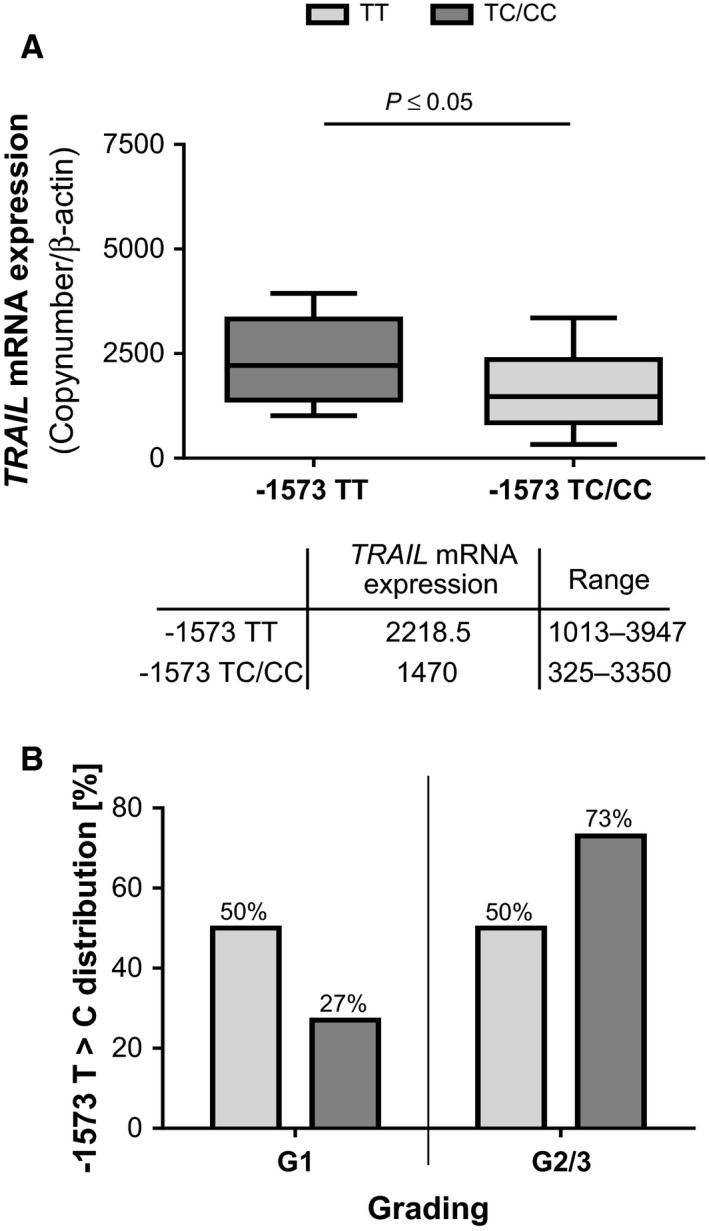
(A) TRAIL mRNA expression in HCC tissue samples in dependence on the ‐1573T>C SNP within the human TRAIL promoter. TRAIL mRNA expression is illustrated as median and range of the TRAIL copy number normalized to *β*‐actin. The frequencies of samples within the two groups are expressed as numbers of entities. The boxplots of TRAIL mRNA expression in patients with the homozygous ‐1573TT genotype or the ‐1573TC/‐1573CC genotypes are displayed as median with 95% coincidence interval. The statistical analysis was performed with the Mann–Whitney *U* Test with a significance level of *P *≤* *0.05. (B) The correlation between the ‐1573T>C genotype distribution and the tumor grade is displayed as ratio of the ‐1573TT or ‐1573TC/TT genotype distribution within patients subdivided into tumor grades. The statistical analysis was performed with the chi‐squared test with Pearson approximation and a significance level of *P *≤* *0.05. T, thymine; C, cytosine; HCC, hepatocellular carcinoma; TRAIL, tumor necrosis factor‐related apoptosis‐inducing ligand; SNP, single‐nucleotide polymorphism.

To determine whether the ‐1573T>C SNP in the human TRAIL promoter results in a reduction in TRAIL mRNA expression, we performed correlation analysis of the ‐1573T>C genotype and measured TRAIL expression in HCC tissue. TRAIL mRNA expression was significantly (*P *≤* *0.05) lower in HCC tissues with the ‐1573TC or ‐1573CC genotype than in those with the ‐1573TT genotype, a finding indicating that this sequence region is a prerequisite for TRAIL expression. Thus, TRAIL expression is significantly impaired in HCC tissues with the ‐1573TC or ‐1573CC genotype (Fig. 2A). Furthermore, the ‐1573T>C genotype is correlated with the differentiation grade of the tumor. The ‐1573TC or ‐1573CC genotype was higher in low‐differentiated HCC tissues (G2–3; 73%) than in high‐differentiated HCC tissues (G1; 27%), whereas the homozygous ‐1573TT genotype showed equal frequencies independent on the differentiation grade of the tumor (Fig. 2B).

### Loss‐of‐function SNP in the human TRAIL promoter

**Figure 3:**
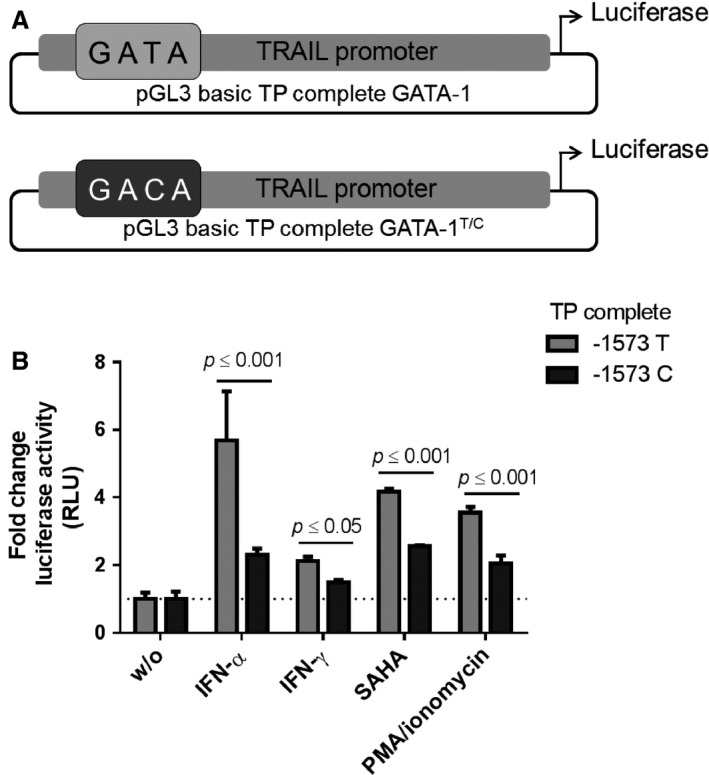
The influence of the ‐1573T>C SNP on the inducibility of the TRAIL promoter. (A) Schematic illustration of the firefly reporter gene constructs with the complete TRAIL promoter region with the ‐1573T or the ‐1573C variation. (B) Huh7 cells were transfected with both TRAIL promoter reporter gene constructs and incubated with several known TRAIL‐inducing agents (500 IU/mL IFN‐*α*, 1000 IU/mL IFN‐*γ*, 100 nmol/L PMA plus 1 *μ*mol/L ionomycin, or 20 *μ*mol/L SAHA) for 6 h. The inducibility of the TRAIL promoter is normalized to the nonstimulated constructs. The statistical analysis was performed with the two‐tailed Student's *t*‐test at a significance level of *P *≤* *0.05. TP, TRAIL promoter; T, thymine; C, cytosine; PMA, phorbol 12‐myristate 13‐acetate; SAHA, N'‐hydroxy‐N‐phenyloctanediamide; RLU, relative light units; w/o, unstimulated control cells; TRAIL, tumor necrosis factor‐related apoptosis‐inducing ligand; SNP, single‐nucleotide polymorphism; PMA, phorbol 12‐myristate 13‐acetate.

To determine whether the ‐1573T>C SNP leads to impairments of TRAIL mRNA expression, we analyzed the inducibility of the TRAIL promoter spanning the in silico GATA‐1‐binding site. Point mutations from T to C were inserted into the binding site at position ‐1573 and a pGL3 luciferase reporter gene vector was generated by inserting either the complete wild‐type (‐1573T) or the mutated (‐1573C) TRAIL promoter sequence (‐1717 to ‐1 upstream of the transcription start site) simulating the different homozygous genotypes (Fig. 3A). Huh7 cells were supplemented with either gene construct and were stimulated with IFN‐*α*, IFN‐*γ*, phorbol 12‐myristate 13‐acetate [PMA] plus ionomycin, suberoylanilide hydroxamic acid [SAHA], as indicated in Figure. 3B. The strongest effect on the ‐1573T TRAIL promoter construct was observable with IFN‐*α*, whereas inducibility of the ‐1573C variant was significantly (*P *≤* *0.01) reduced. Less activity was observed after treatment of the TRAIL promoter with IFN‐*γ*, SAHA, or PMA/ionomycin (Fig. 3B).

### Functionality of the ‐1573T>C SNP in the human TRAIL promoter

**Figure 4:**
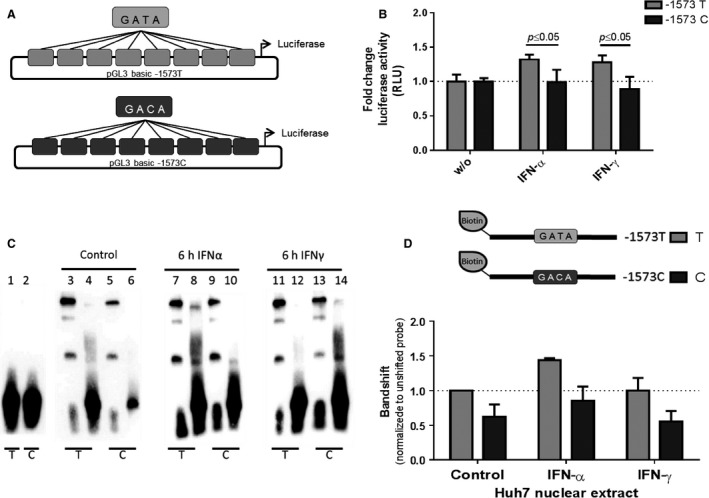
The influence of the ‐1573T>C SNP on the functionality of sequence region. (A) Schematic illustration of the firefly reporter gene constructs with the repetitive sequence around the ‐1573T>C SNP reflecting the ‐1573T or the ‐1573C genotype. (B) For reporter gene assays, Huh7 cells were transfected with both luciferase reporter gene constructs and incubated with 500 IU/mL IFN‐*α* or 1000 IU/mL IFN‐*γ* for 6 h. The inducibility of the TRAIL promoter is normalized to the nonstimulated constructs. The statistical analysis was performed with the two‐tailed Student's *t*‐test at a significance level of *P *≤* *0.05. (C) Results of EMSA bandshift assays with the biotin‐labeled oligonucleotides for the ‐1573T and the ‐1573C sequence of the human TRAIL promoter with nuclear extracts from Huh7 cells, untreated or stimulated with 500 IU/mL IFN‐*α* or 1000 IU/mL IFN‐*γ* for 6 h. Lane 1 + 2: ‐1573T or ‐1573C probe without nuclear extracts (schematic illustration Fig. 5D); Lane 3, ‐1573T probe with nuclear extracts of untreated Huh7 cells; Lane 4, ‐1573T probe with nuclear extracts of untreated Huh7 cells and 100‐fold excess of the unlabeled competitor oligonucleotide; Lane 5, ‐1573C probe with nuclear extracts of untreated Huh7 cells; Lane 6, ‐1573C probe with nuclear extracts of untreated Huh7 cells and 100‐fold excess of the unlabeled competitor oligonucleotide; Lane 7, ‐1573T probe with nuclear extracts of Huh7 cells treated with IFN‐*α*; Lane 8, ‐1573T probe with nuclear extracts of Huh7 cells treated with IFN‐*α* and 100‐fold excess of the unlabeled competitor oligonucleotide; Lane 9, ‐1573C probe with nuclear extracts of Huh7 cells treated with IFN‐*α*; Lane 10, ‐1573C probe with nuclear extracts of Huh7 cells treated with IFN‐*α* and 100‐fold excess of the unlabeled competitor oligonucleotide; Lane 11, ‐1573T probe with nuclear extracts of Huh7 cells treated with IFN‐*γ*; Lane 12, ‐1573T probe with nuclear extracts of Huh7 cells treated with IFN‐*γ* and 100‐fold excess of the unlabeled competitor oligonucleotide; Lane 13, ‐1573C probe with nuclear extracts of Huh7 cells treated with IFN‐*γ*; Lane 14, ‐1573C probe with nuclear extracts of Huh7 cells treated with IFN‐*γ* and 100‐fold excess of the unlabeled competitor oligonucleotide. (D) Fold change of the bandshifts with ‐1573T or ‐1573C probe and nuclear extracts of untreated Huh7 cells or after treatment with IFN‐*α* or IFN‐*γ*. The bar graph represents the average and standard deviations of three independent experiments. T, thymine; C, cytosine; RLU, relative light units; w/o, unstimulated control cells. EMSA, electron mobility shift assay; TRAIL, tumor necrosis factor‐related apoptosis‐inducing ligand; SNP, single‐nucleotide polymorphism.

To confirm the importance of the ‐1573T>C genotype for the regulation of TRAIL expression, we inserted synthetic DNA fragments with a repetitive sequence spanning the region around the ‐1573T>C SNP, either with the ‐1573T or the ‐1573C genotype into a pGL3 luciferase reporter gene vector (Fig. 4A). In Huh7 hepatoma cells, the constructs with the ‐1573C sequence exhibited significantly (*P *≤* *0.05) less activity after stimulation than those with the ‐1573T sequence harboring the in silico GATA‐1‐binding site (Fig. 4B).

### ‐1573C sequence variation affects promoter‐binding affinity

To analyze the influence of the ‐1573C genotype on the transcription factor binding site pattern within the human TRAIL promoter, we performed EMSAs with oligonucleotides representing the TRAIL promoter and featuring either the ‐1573T with the intact binding site or the ‐1573C genotype, labeled with the 5′‐end attachment of biotin (Fig. 4D). We analyzed nuclear extract of Huh7 cells either untreated or treated with IFN‐*α* or IFN‐*γ* to determine the binding patterns of the of the TRAIL promoter in dependence on the genotype of the ‐1573T>C SNP.

Huh7 nuclear protein extracts which were incubated with the probe containing the ‐1573T genotype exhibited a specific binding pattern with three separate shifts (Fig. 4C, lines 3, 7, 11). The binding pattern of the ‐1573C probe showed not only distinct differences from the ‐1573T probe but also between the treatments applied to the cells. Figure 4D displays the intensity of the respective bandshifts normalized to the unshifted probe.

The binding pattern of the ‐1573C probe showed less intense band shifts compared to the ‐1573T probe (Fig. 4C, lines 5, 9, 13), independent from the treatment applied. In addition, upon treatment with IFN‐*α* or IFN‐*γ*, only the intensity of the band shifts of the ‐1573T probe increased (Fig. 4C*,* lines 3, 7, 11). These results reveal not only an enhanced binding of the ‐1573T probe after treatment of the cells with IFNs but also impaired binding of the ‐1573C probe with or without IFN treatment (Fig. 4D). In order to determine whether the in silico GATA‐1‐binding sequence refers to a GATA‐1‐binding element in vivo, supershift assays were performed that confirmed the impact of the ‐1573T>C SNP on TRAIL promoter functionality. However, we could not induce a supershift with a specific GATA‐1 antibody (data not shown), revealing that GATA‐1 does not directly bind to the promoter in vivo.

### Impaired TRAIL expression supports tumor cell migration

**Figure 5:**
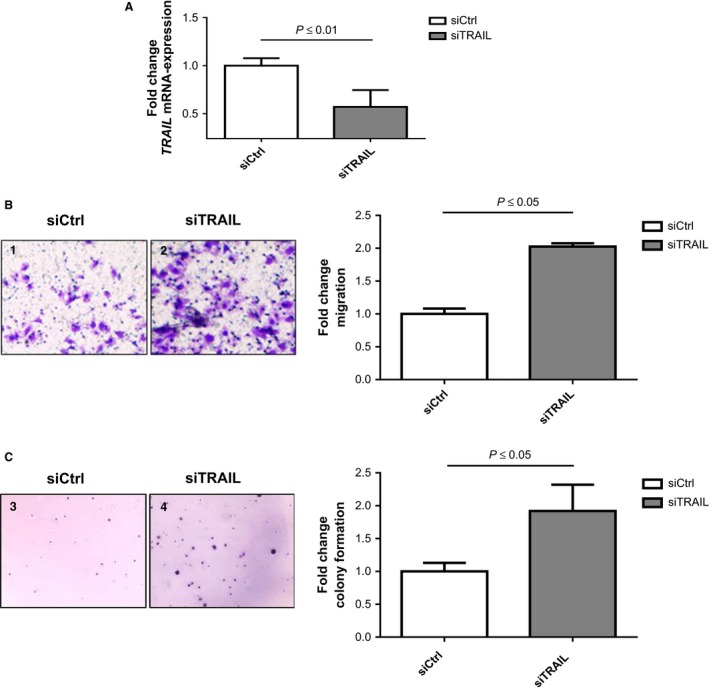
The influence of TRAIL on the migration and colony‐formation potential of hepatoma cells. (A) Huh7 cells were transfected with either TRAIL‐specific (siTRAIL) or control (siCtrl) siRNA for 48 h and analyzed for TRAIL mRNA expression. The TRAIL mRNA expression is illustrated as copy number normalized to *β*‐actin. (B) Huh7 cells transfected with either TRAIL‐specific (siTRAIL) or control (siCtrl) siRNA for 48 h were analyzed for their migration potential in Boyden chamber assays. (C) Huh7 cells transfected with TRAIL‐specific (siTRAIL) siRNA for 48 h were analyzed for their colony‐formation potential and compared with cells transfected with control (siCtrl) siRNA for 48 h. The statistical analyses were performed with the Student's *t*‐test at a significance level of *P *≤* *0.05. siCtrl, control siRNA; siTRAIL, TRAIL‐specific siRNA. TRAIL, tumor necrosis factor‐related apoptosis‐inducing ligand; siRNA, small interfering RNA.

To further clarify the functional role of TRAIL in liver tumor cells, we performed RNA interference with subsequent analysis of the migration and colony‐formation potential in hepatoma cells. TRAIL RNA silencing in Huh7 cells resulted in a significant (*P *≤* *0.01) reduction in TRAIL mRNA expression (Fig. 5A). Migration was significantly (*P *≤* *0.05) higher in those cells with impaired TRAIL expression than in cells with normal WT TRAIL expression (Fig. 5B). In addition, the colony‐formation potential of Huh7 cells increased significantly (*P *≤* *0.05) after TRAIL mRNA expression was reduced. Huh7 cells with reduced TRAIL expression developed approximately twice as many colonies as Huh7 cells with WT TRAIL expression (Fig. 5C).

## Discussion

Hepatocellular carcinoma is characterized by a vast molecular heterogeneity; the path of tumor development is paved with a magnitude of genetic changes often complicated by individual combinations or multiallelic deletions and chromosomal losses 25, 26, 27, 28, 29, 30, 31. A body of evidence has described TRAIL to be essentially involved in HCC pathophysiology.

We have previously shown that TRAIL expression is impaired in a high number of HCCs and is, in addition, associated with the size, spread, and differentiation of the tumor 18. The findings presented here elucidate further, a molecular mechanism by which TRAIL regulation may be impaired in HCC pathology.

PC‐based sequence comparisons of 93 human HCC samples revealed in 44% of the tumors, a sequence variation at position ‐1573 upstream of the transcription start site. Deeper sequence analysis identified this sequence variation as ‐1573T>C SNP. Most (56%) of the patients displayed the ‐1573TT homozygous genotype, 28% the heterozygous ‐1573TC genotype, and only 16% exhibited the ‐1573CC genotype (Fig. 1C). The correlation of the genotype distribution with the tumor grading (Fig. 2B) as well as the absence of a tumor‐specific mutation supports the assumption that the ‐1573T>C SNP is a factor predisposing cells to HCC development in the context of other oncogenic factors. In silico, only the ‐1573T genotype refers to a GATA‐1‐binding sequence what prompted us to assume a loss‐of‐function mutation in this sequence due to the ‐1573C genotype. Indeed, nucleotide exchange from T to C leads to a loss of function of the human TRAIL promoter in reporter gene assays and demonstrates the indispensability of this element for the TRAIL promoter activity, particularly the inducibility of TRAIL in response to apoptosis‐inducing cytokines (Fig. 3). However, no relation to GATA‐1 was observable in vitro supposing that the in silico suggestion of a GATA‐1 site in this position does not hold true in vivo. However, a more in‐depth analysis of the TRAIL promoter region spanning this ‐1573T>C SNP confirms the influence of this binding site on TRAIL regulation. Only the ‐1573T genotype is inducible with both IFN‐*α* and IFN‐*γ* (Fig. 4B). EMSAs confirmed the functional relevance of the ‐1573T>C SNP by demonstrating the diminished binding of the ‐1573C genotype (Fig. 4D). Thus, the nucleotide exchange from T to C at position ‐1573 indicates a loss‐of‐function mutation within the human TRAIL promoter with consecutive impairment of TRAIL expression. The loss‐of‐function mutation may result in an advantage for HCC development and growth by impairing TRAIL regulation and expression. One consequence of impaired TRAIL expression is the facilitation of unrestricted proliferation, which fosters the development and outgrowth of the tumor. These considerations are supported by our findings showing the correlation between TRAIL expression and HCC progression, as demonstrated by tumor size, spread, and grade (18). The ‐1573T>C SNP results in a reduction in TRAIL mRNA expression (Fig. 2A), that probably enhances the tissue's susceptibility to tumor development and progression. These considerations are supported by the findings of studies demonstrating that apoptosis is impaired by dysregulation of TRAIL and TRAIL receptors in several other tumor entities 32, 33 and that TRAIL receptors influence the pathogenesis of several liver diseases. Moreover, compared to hepatoma cells with normal TRAIL expression, hepatoma cells with decreased TRAIL expression exhibit a considerable increase in migration and in colony‐formation potential (Fig. 5), a finding indicating that a loss of TRAIL expression impairs the balance between cell death and differentiation. These observations are supported by studies demonstrating enhanced formation of metastasis in TRAIL‐deficient mice 34, a finding confirmed by Grosse‐Wilde et al., who showed that not only the loss of TRAIL but even the loss of the TRAIL receptor leads to a decrease in apoptosis and further contributes to an enhancement in metastasis formation 17. Considering the consequences of impaired TRAIL expression and regulation for HCC development and growth, it would be tempting to exploit these findings for therapeutic respects. The implication of TRAIL in liver diseases has already been analyzed 35. Additionally, the safety of soluble TRAIL was demonstrated for therapy, in vivo, but also showed that the application of TRAIL decreases tumor progression by suppressing tumor xenografts 36, 37 and supports the consideration that patients with reduced TRAIL expression may benefit from therapeutic agents with TRAIL‐inducing effects 38, 39, 40. Our findings underline the relevance of TRAIL expression and promoter mutation in the development and progression of HCC. The confirmation of the herein presented correlation of the ‐1573T>C SNP with disease severity and progression will be of future interest. Thereby, determination of the sensitivity and specificity as well as the extension of the correlation analysis concerning epidemiological parameters shall verify the ‐1573T>C SNP as a useful tool in a clinical setting, either for monitoring of these patients at risk in terms of HCC development or recurrence after surgical resection and liver transplantation.

## Conflict of Interest

The contributing authors declare no conflicts of interest with the content of the manuscript.
